# Recurrent orbital bone sub-periosteal hematoma in sickle cell disease: a case study

**DOI:** 10.1186/s12886-018-0884-1

**Published:** 2018-08-28

**Authors:** Abdulhamid Alghamdi

**Affiliations:** Department Of Ophthalmology, Faculty of Medicine, Taif University, Taif, Saudi Arabia

**Keywords:** High altitude, Orbital bone infarction, Orbital bone sub-periosteal hematoma, Orbital compression, Proptosis, SCD

## Abstract

**Background:**

Sickle cell disease is a common inherited hemoglobinopathy and is associated with high morbidity and mortality. Vaso-occlusive crises commonly occur in individuals with SCD that results in high morbidity due to end-organ ischemia and infarction. These include splenic infarction, pulmonary involvement, acute chest syndrome, and orbital compression syndrome. Ocular manifestations of SCD include anterior segment ischemia, secondary glaucoma, angoid streaks, retinopathy, and retinal artery occlusion. Commonly reported causes for the incidence of sickle cell disease are extreme temperatures, wind speed, and rainfall. This study has conducted an investigation of recurrent orbital bone sub-periosteal hematoma in a sickle cell patient that was exposed to high altitude areas.

**Case presentation:**

A 12-year-old boy with SCD developed a recurrent sudden periorbital pain and swelling during a visit to high altitude area. The family reported two similar attacks previously. The patient recovered completely with timely initiated conservative treatment. The case study is about homozygous SCD with previous history of similar attack of painful periorbital swelling that resolved after conservative management. This condition was associated with proptosis, diplopia, and restriction of eye movement. Magnetic resonance imaging of the orbits showed right orbital roof subperiosteal mass adjacent to the orbital wall, which was identified as a subperiosteal haematoma, inducing proptosis. The patient was discharged after 7 days with follow up.

**Conclusions:**

Infarction of orbital bones during vaso-occlusive crises in SCD presented acutely with a rapidly progressive painful periorbital swelling. Hematomas frequently complicate the condition, along with the inflammatory swelling that may lead to the orbital compression syndrome. The condition is sight-threatening and necessitates prompt diagnosis along with appropriate management. This condition mandates prompt initiation of conservative treatment and close monitoring of the optic nerve functions to prevent permanent visual loss in young patients.

## Background

Sickle Cell Disease (SCD) is the most commonly inherited hemoglobinopathy worldwide and has resulted in the increase of global health burden [1]. SCD is an autosomal recessive disorder, characterized by production of abnormal hemoglobin S, and is associated with high morbidity and mortality and is a relatively common genetic disorder across the world [2]. A pre-dominant feature of SCD is the occurrence of vaso-occlusive crises. A vaso-occlusive crisis affects all organ systems and are responsible for high morbidity and early mortality due to end-organ ischemia and infarction. These conditions are combined with the downstream effects of hemolysis due to red blood cell sickling. The clinical presentation of the end-organ vaso-occlusive crisis may mimic many clinical conditions, which creates a diagnostic dilemma. Recent reports of end-organ ischemia include splenic infarction [3], pulmonary involvement, acute chest syndrome [4], and orbital compression syndrome [5, 6]. Ocular manifestations of SCD include anterior segment ischemia, secondary glaucoma, angoid streaks, retinopathy, and retinal artery occlusion. Autoinfarction of the orbital bones has been reported rarely. It can lead to acute proptosis, periorbital pain, limited motility, potentially compressive optic neuropathy, and orbital compression syndrome (OCS) [7, 8].

Commonly reported causes for the incidence of SCD are inclusive of extreme temperatures, wind speed, and rainfall [9]. It was discussed that these reported environmental factors significantly predispose sickle cell patients to acute vaso-occlusive crises. Although, a few studies have highlighted the relatively higher incidence of SCD in individuals living in high-altitude or mountainous areas [10]; the role that high altitude plays in increasing the risk of incidence of sickling cell crises has not yet been heavily explored. Earlier studies have highlighted cases of sickling cell crisis incidence in individuals flying at high-altitude areas in unpressurized aircrafts [11]. The incidence of altitude-induced hypoxemia leading to painful sickling crises was explored in an additional study that suggested that there was high risk presented to sickle cell patients when residing in high-mountainous regions [12]. This finding was corroborated by additional studies that highlighted the higher incidence of splenic infections in sickle cell patients following altitude-induced hypoxia [13]. However, the link between other types of infarction in sickle cell patients and high altitudes has not yet been established. Therefore, this study has conducted an investigation of recurrent orbital bone sub-periosteal hematoma in a sickle cell patient that was exposed to high altitude areas.

## Case presentation

**Fig. 1:**
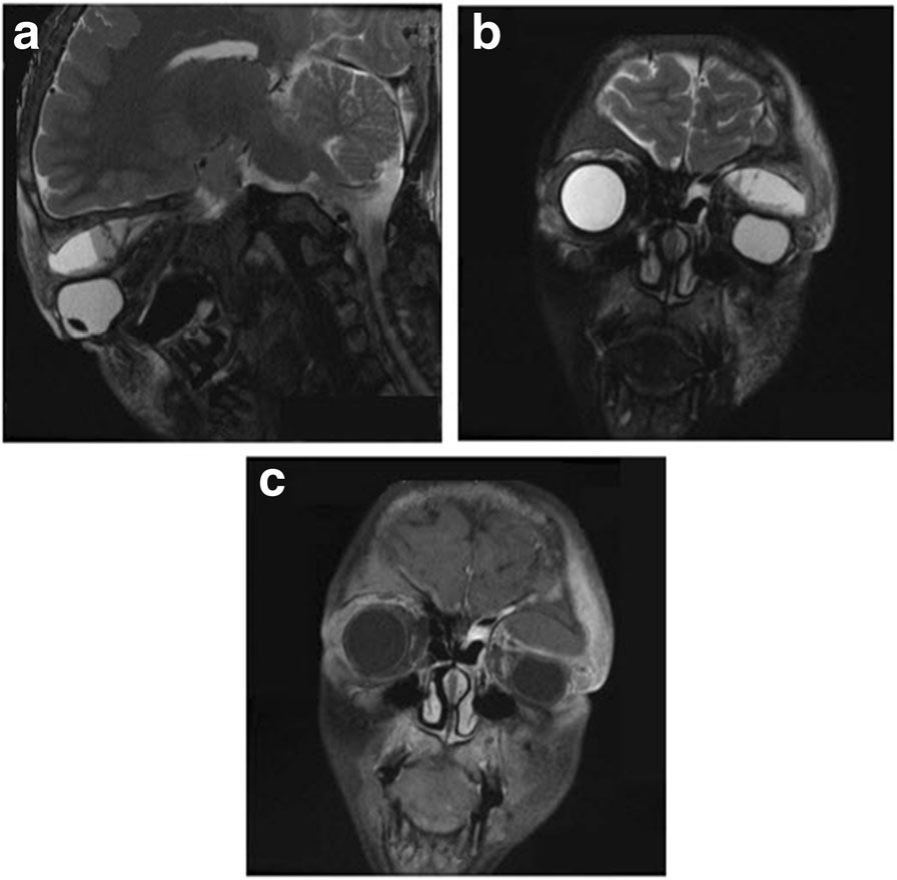
**a** Coronal T2 Fat Suppression; (**b**) Sagittal T2 Fat Suppression; (**c**) Coronal T1Fat Suppression

A 12-year-old boy with SCD was presented with fever, periocular pain, and diplopia after returning from Taif, Jeddah. Taif (means “encompassing”) is located in the Hejaz Mountains of Saudi Arabia. It is considered as a high-altitude area because it is 6000 ft above the sea level [14]. The patient had a previous history of similar attacks that resolved after conservative management at another hospital in the same city few years ago (Fig. 1).

On admission, the patient looked sick, drowsy, and pale. The temperature of patient was 38.2 °C, heart rate was 115/min, respiratory rate was 25/min, blood pressure was 100/65, and oxygen saturation was 90% on room air. The patient weighted 38 kg. Ocular examination showed right eyelid edema, peri-ocular soft tissue swelling, proptosis, and limitation in elevation of the right eye. On admission, the visual acuity of right eye was 20/30 and left eye was 20/20. Color vision was evaluated using the color plates that came out to be normal. The pupils were equal in size and reactive to light. Swinging light Reflex showed normal reaction of both pupils. There was no afferent pupillary light reflex defect (APD). The intra-ocular pressure was normal in both eyes. Fundus examination revealed normal disc, blood vessels, and macula. A complete systemic evaluation was conducted.

The systemic evaluation revealed hemolytic anemia, thrombocytopenia, stable coagulation profile, and negative blood culture. Laboratory results showed hemoglobin level of 89 g/ L, mean cell volume was 84.2 FL, white blood cell count was 24.04X109/L with neutrophils 21.81X109/L, and mean platelets volume 10.30 FL. Serum bilirubin was measured to be 95.5 mmol/L, albumin was 26 g/L, blood urea was 3.8 mmol/L, and serum creatinine was 39 mmol/L. The erythrocyte sedimentation rate was 40 mm/h (normal < 15 mm/h), and C-reactive protein was 8.2 mg/dL (normal < 0.5 mg/dL) (Table 1).

**Table 1:** Summary of lab results

Test	Result	Unit	N. Range
White Blood Cell Count	24.04	X 10^9^/L	4–10
Neutrophils Count	21.81	X 10^9^/L	2–9
Red Blood Cell Count	3.03	X 10^12^/L	4.3–5.5
Hematocrit	25.5	%	36–46
Mean Cell Volume	84.2	FL	80–97
Mean Cell Hemoglobin	29.4	PG	27–33
Hemoglobin Consentration	89	G/L	120–150
Platelet Count	225	X 10^9^/L	150–400
Mean Platlet Volume	9.5	FL	7–11
Erythrocyte Sedimentation Rate	40	mm/h	00–15

**Fig. 2:**
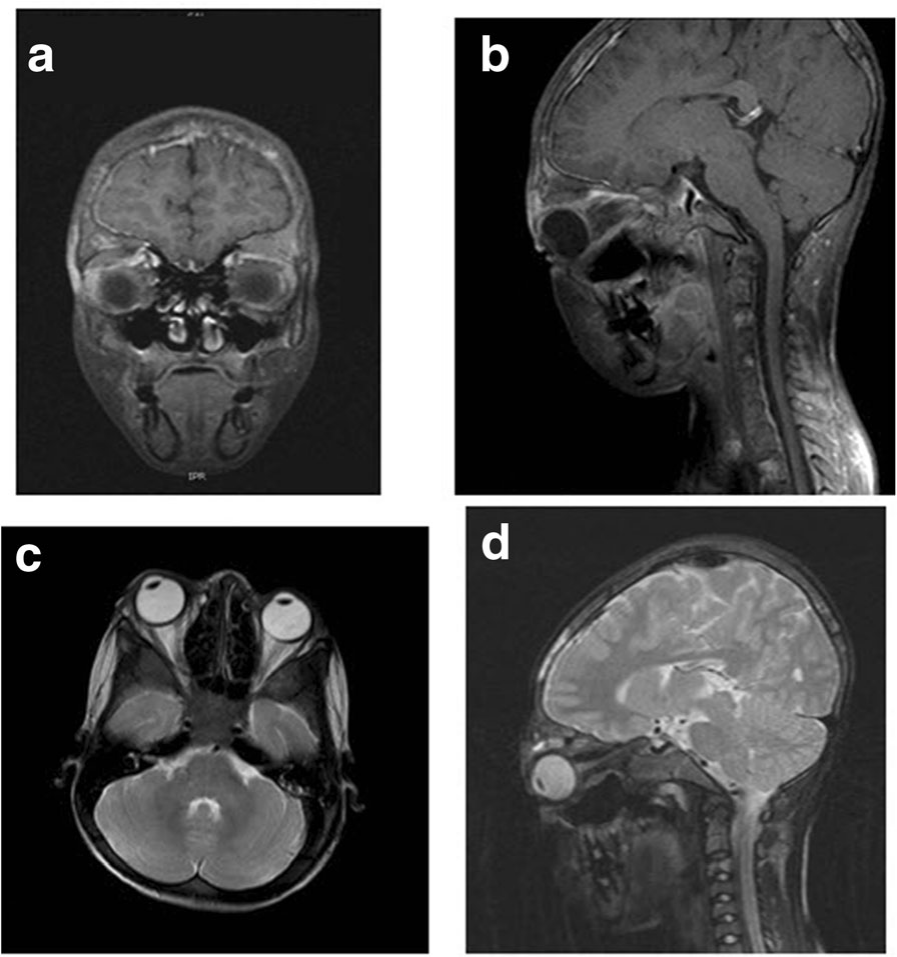
**a** Sagittal T1 FS Post Contrast; (**b**) Faint marginal enhancement of the superior sub periosteal orbital roof frontal bone; (**c**) Sagittal T2 Fat Suppression; (**d**) Axial T2 Fat Suppression

The coagulation parameters revealed a prothrombin time (PT) of 14 S (normal 10–12.8), International Normalization Ratio (INR) 1.2 (normal 0.9–1.2) and activated partial prothrombin time (aPTT) 33.4 S (normal 25.3–38.4). Hemoglobin electrophoresis showed HbS 58%, HbA 36%, HbF 2%, and HbA2 4% (consistent with sickle b thalassemia). Urine analysis was normal, and the culture report was negative. Magnetic Resonance Imaging (MRI) of the right orbit demonstrated peri-orbital edema and a mass adjacent to the right orbital wall. This condition was identified as a superior subperiosteal haematoma with evidence of orbital bone and bone marrow abnormal signals consistent with orbital wall infarction (Fig. 2).

**Fig. 3:**
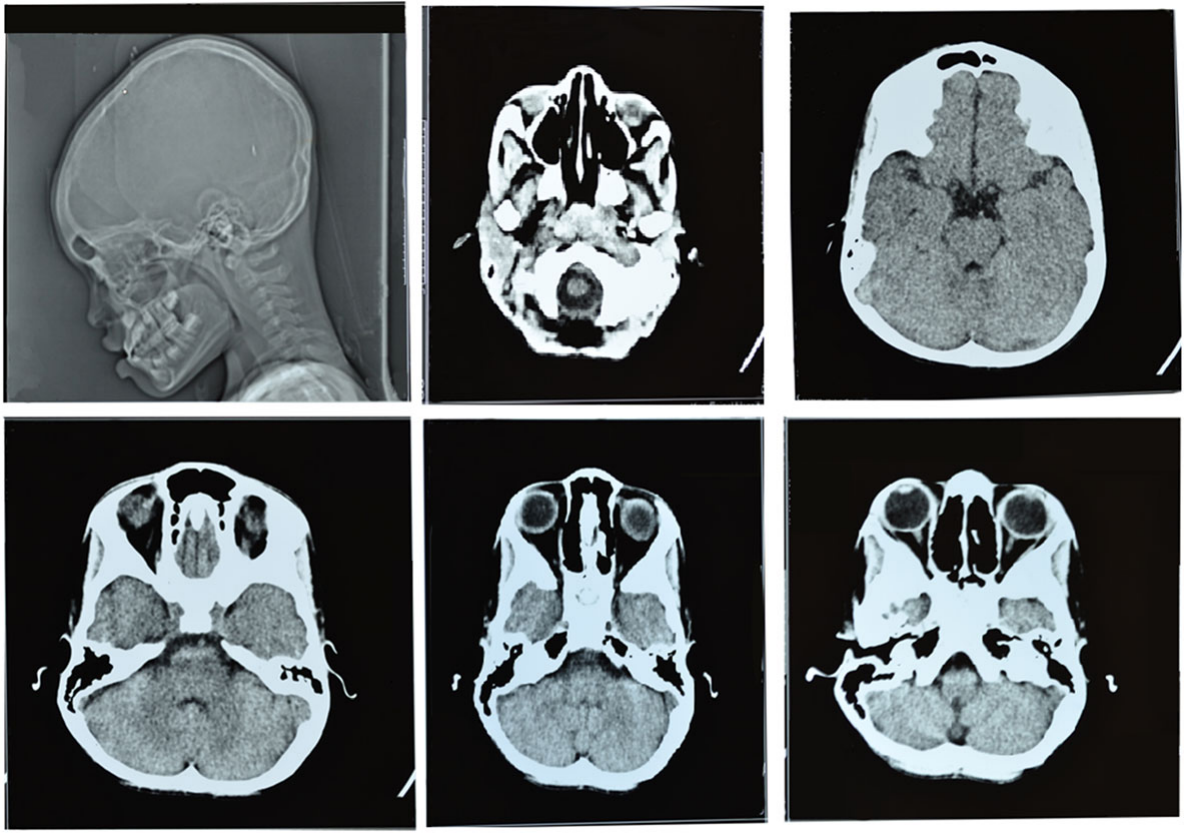
CT scan images of subperiosteal orbital bone abnormality

The bone abnormality was further investigated to explore the possibility of the presence of primary or metastatic tumors that are susceptible to bleeding. Therefore, CT-imagery was utilized to explore this area of interest. However, as may be seen from the CT images, there was no evidence of primary or metastatic bone tumors (Fig. 3).

The patient received intravenous fluids, analgesics, broad spectrum antibiotics, and pulse methylprednisolone immediately. The patient responded well to medical management with complete recovery and was discharged after the condition was stabilized. This case has highlighted the importance of considering orbital wall infarction in the differential diagnosis of orbitopathy among the patients with SCD, along with osteomyelitis and orbital abscess. Careful evaluation, diagnosis, and the prompt initiation of the appropriate supportive care are highly recommended in order to prevent permanent visual loss.

## Discussion and conclusion

SCD is the most common inherited hemoglobinopathy, which results from a point mutation (GAG/GTG) in exon 1 of the β globin gene. It results in the substitution of glutamic acid by valine at position 6 of the β globin polypeptide chain. An abnormal hemoglobin S molecule is formed, which polymerizes under the conditions of hypoxia and acidosis. It will cause the red blood cell to become sickle in shape and rigid. These cells have short lifespan (chronic hemolysis) and tend to block capillaries (vaso-occlusion), which explains almost all clinical manifestations of SCD [15].

Orbital compression syndrome (OCS) is an acute condition characterized by eyelid edema, proptosis, periorbital pain, and restriction of extra-ocular motility, with or without decreased visual acuity. The main mechanism for the development of OCS is orbital bone infarction with subsequent inflammatory response that can rapidly spread to the orbit resulting in orbital pain and proptosis [6]. A unique feature of orbital wall infarction in SCD is the formation of hematomas, which may be orbital (subperiosteal) or intracranial (epidural) [8]. Several mechanisms have been suggested for the development of subperiosteal hemorrhage; such as extravasation of blood from necrotized vessel walls, underlying bleeding diathesis, and minor trauma [16]. Presumed cause of orbital hematoma in the present case is orbital infarction that was evident by abnormal frontal bone and bone marrow heterogeneous signals adjacent to the formation of hematomas.

Orbital wall infarction typically occurs among young patients because there is more marrow space in the orbital bone among children, unlike the adults [16]. The mean age of presentation in the reported cases was 13 years, with the youngest reported age at 2 years. It affects almost twice as many males as females. The OCS has wide spectrum of manifestations ranging from its mild form which constitutes pain and eyelid edema to the most severe of bilateral proptosis, chemosis, limited ocular motility, and vision loss. Fever at presentation occurs in almost all cases, and associated pain crises elsewhere were seen in more than two thirds of the cases. Bilateral orbital involvement was reported in one third of cases [7]. The differential diagnosis of acute periorbital pain and swelling with or without other manifestations of OCS in patients with SCD include orbital cellulitis, orbital abscess, pre-orbital cellulitis, osteomyelitis of orbital bone, orbital tumor and orbital bone infarction.

It is crucial and challenging to differentiate between orbital bone infarction and orbital osteomyelitis, and the presence of Leukocytosis and elevated erythrocyte sedimentation rate as C-reactive protein can occur in both bone infection and infarction [17]. Bone marrow abnormalities are well documented by MRI as well as orbital soft-tissue swelling, sub-periosteal hemorrhage, and fluid collections. MRI showed the location, size, and effects of the hematoma on the adjacent orbital structures including the optic nerve. The presence of abnormal MRI signals indicated acute inflammatory process in the area of the orbital roof and bone marrow of the frontal bone in the present case. Bone marrow scan confirms marrow infarction in 95% of cases by demonstrating decreased tracer uptake; whereas, in infection the uptake is normal. MRI was the imaging modality of choice for evaluating OCS in majority of the reported cases. [17].

The present case is unique because the condition is recurrent in the frontal bone, triggered by visit to a high-altitude city. Similar studies pertaining to infarctions in SCD at high-altitude areas have been conducted; however, this is the first study to conduct an exploration of orbital bone infarction in SCD at high-altitude areas [10, 13]. However, previously OCS attacks had resolved completely with conservative measures. Administration of intravenous corticosteroids may relieve orbital pressure, caused by inflammatory component of orbital bone infarction. Concomitant antibiotic coverage is advisable as it is often difficult to clinically differentiate osteomyelitis from bone infarction. Surgical exploration and evacuation of hematoma is warranted to prevent vision loss and speedy recovery, if there are signs of optic nerve dysfunction. Majority of the reported patients recovered with medical therapy, and around one fifth of these patients required surgical interventions. Two of the reported cases ended with permanent visual loss because of optic nerve compression [8]. Clinical optic nerve dysfunction, significant eye globe displacement, and thinning and touristy of optic nerve on MRI were considered as poor prognostic signs. Surgical intervention and draining of hematomas should be performed, once the patient demonstrates signs of optic nerve compression that poorly respond to conservative management.

The diagnosis of OCS should be considered, when patients with SCD complain of proptosis, decreased extra ocular motility, eyelid edema, and optic neuropathy. Children with SCD are susceptible to infections, and empirical use of broad spectrum antibiotics should be considered, if infectious process is suspected. Imaging techniques are essential in the evaluation of patients, suffering osteomyelitis and orbital abscess. Majority of cases resolve with conservative treatment that includes hydration and oxygen to reverse the vaso-occlusive crisis and use of systemic steroids under antibiotic cover. Critical evaluation and prompt surgical intervention is needed to prevent loss of vision, if evidence of optic nerve dysfunction or large hematoma is present.

## Abbreviations

APD: Afferent pupillary light reflex defect
MRI: Magnetic Resonance Imaging
OCS: Orbital compression syndrome
SCD: Sickle cell disease
